# Maintenance of genome integrity by the late-acting cytoplasmic iron-sulfur assembly (CIA) complex

**DOI:** 10.3389/fgene.2023.1152398

**Published:** 2023-03-08

**Authors:** M. S. Petronek, B. G. Allen

**Affiliations:** Division of Free Radical and Radiation Biology, Department of Radiation Oncology, University of Iowa, Iowa City, IA, United States

**Keywords:** iron metabolism, Fe-S biogenesis, genomic integrity, DNA metabolism, CIA complex

## Abstract

Iron-sulfur (Fe-S) clusters are unique, redox-active co-factors ubiquitous throughout cellular metabolism. Fe-S cluster synthesis, trafficking, and coordination result from highly coordinated, evolutionarily conserved biosynthetic processes. The initial Fe-S cluster synthesis occurs within the mitochondria; however, the maturation of Fe-S clusters culminating in their ultimate insertion into appropriate cytosolic/nuclear proteins is coordinated by a late-acting cytosolic iron-sulfur assembly (CIA) complex in the cytosol. Several nuclear proteins involved in DNA replication and repair interact with the CIA complex and contain Fe-S clusters necessary for proper enzymatic activity. Moreover, it is currently hypothesized that the late-acting CIA complex regulates the maintenance of genome integrity and is an integral feature of DNA metabolism. This review describes the late-acting CIA complex and several [4Fe-4S] DNA metabolic enzymes associated with maintaining genome stability.

## Introduction

Iron-sulfur (Fe-S) clusters are evolutionarily conserved co-factors ubiquitous throughout biology. Fe-S clusters and Fe-S biogenesis are largely conserved throughout prokaryotic and eukaryotic systems as mammalian Fe-S biogenesis enzymes have many shared features with bacteria that have been proposed to be a central cellular feature passed down from alphaproteobacterium (Freibert et al., 2017). Fe-S clusters exist inside proteins as either a [2Fe-2S]^+^, [4Fe-4S]^2+^, or [3Fe-4S]^+^ clusters. Each cluster type is typically specific to the enzymatic function of either 1) electron transfer, 2) enzyme catalysis, or 3) regulation of biological processes (Saha et al., 2018). Fe-S cluster-containing enzymes control a wide array of cellular functions, most notably mitochondrial respiration by the electron transport chain (complex I, II, and II) (Read et al., 2021). However, Fe-S cluster enzymes and Fe-S metabolism are involved in several other cellular processes including lipid metabolism, protein translation, and DNA replication (Fuss et al., 2015; Mettert and Kiley, 2015; Braymer and Lill, 2017; Crooks et al., 2018; Shi et al., 2021). Thus, synthesizing Fe-S clusters is critical in maintaining global cellular homeostasis, underscored by the number of Fe-S-containing enzymes involved in maintaining genome integrity.

The maintenance of genomic integrity is a critical feature of cellular homeostasis by facilitating stable DNA replication with a low mutational burden and cell survival under stressed conditions. Due to its importance, there exists a highly coordinated DNA metabolic network consisting of multi-faceted DNA polymerases that not only replicate DNA during the S-phase of the cell cycle but also facilitate DNA repair (Fuss et al., 2015; Shi et al., 2021). At each level, these DNA metabolic features can be altered by iron either chemically or metabolically. Thus, iron metabolism should be considered an integral component of DNA metabolism.

Due to its ability to catalyze oxidation reactions through either Fenton chemistry or reactions with molecular oxygen, iron is considered a chemical catalyst for site-specific DNA damage (Wardman and Candeias, 1996; Qian and Buettner, 1999; Kruszewski, 2003). For example, ferrous and ferric iron can enhance both single and double-stranded DNA damage associated with the radiolysis of H_2_O (Ambroz et al., 2001). However, there are a large number of Fe-S-containing enzymes within the DNA metabolic system (Fuss et al., 2015). Since the discovery that MMS19 coordinates with DNA metabolic enzymes, a foundational and mechanistic link between the late-acting CIA complex and DNA metabolism has been established (Gari et al., 2012; Stehling et al., 2012). Therefore, it can be postulated that Fe-S biogenesis in total plays a critical role in DNA metabolism and the maintenance of genome integrity. In this review, we provide an overview of the insertion of a [4Fe-4S]^2+^cluster into cytosolic and nuclear apo-proteins via the late-acting CIA complex, describe the role of [4Fe-4S] cluster-containing enzymes in DNA metabolism, and discuss the possible implications of this connection from the systems biology of disease perspective.

## Components of the late-acting cytoplasmic iron-sulfur assembly (CIA) complex

The first step of Fe-S biogenesis is *de novo* [2Fe-2S]^+^ synthesis that occurs on the inner mitochondrial membrane using ISCU as a scaffold. Following the completion of the [2Fe-2S]^+^ cluster synthesis on the ISCU scaffold, the co-factor is trafficked to the late-acting CIA complex for insertion into the appropriate apo-proteins. This is a highly coordinated process that encompasses enzymes required for [2Fe-2S]^+^ synthesis on ISCU (e.g., NFS1) along with [2Fe-2S]^+^ trafficking/ISCU recycling (e.g., HSC20/HSPA9). Following the formation and trafficking of [4Fe-4S]^2+^ clusters, insertion into appropriate intra- and extramitochondrial apo-proteins occur in multiple distinct pathways. The primary focus of this review is on the late-acting CIA complex. More extensive detail regarding *de novo* Fe-S biogenesis can be found in (Petronek et al., 2021).

### Extramitochondrial [4Fe-4S]^2+^ formation and insertion into apo-proteins

**FIGURE 1:**
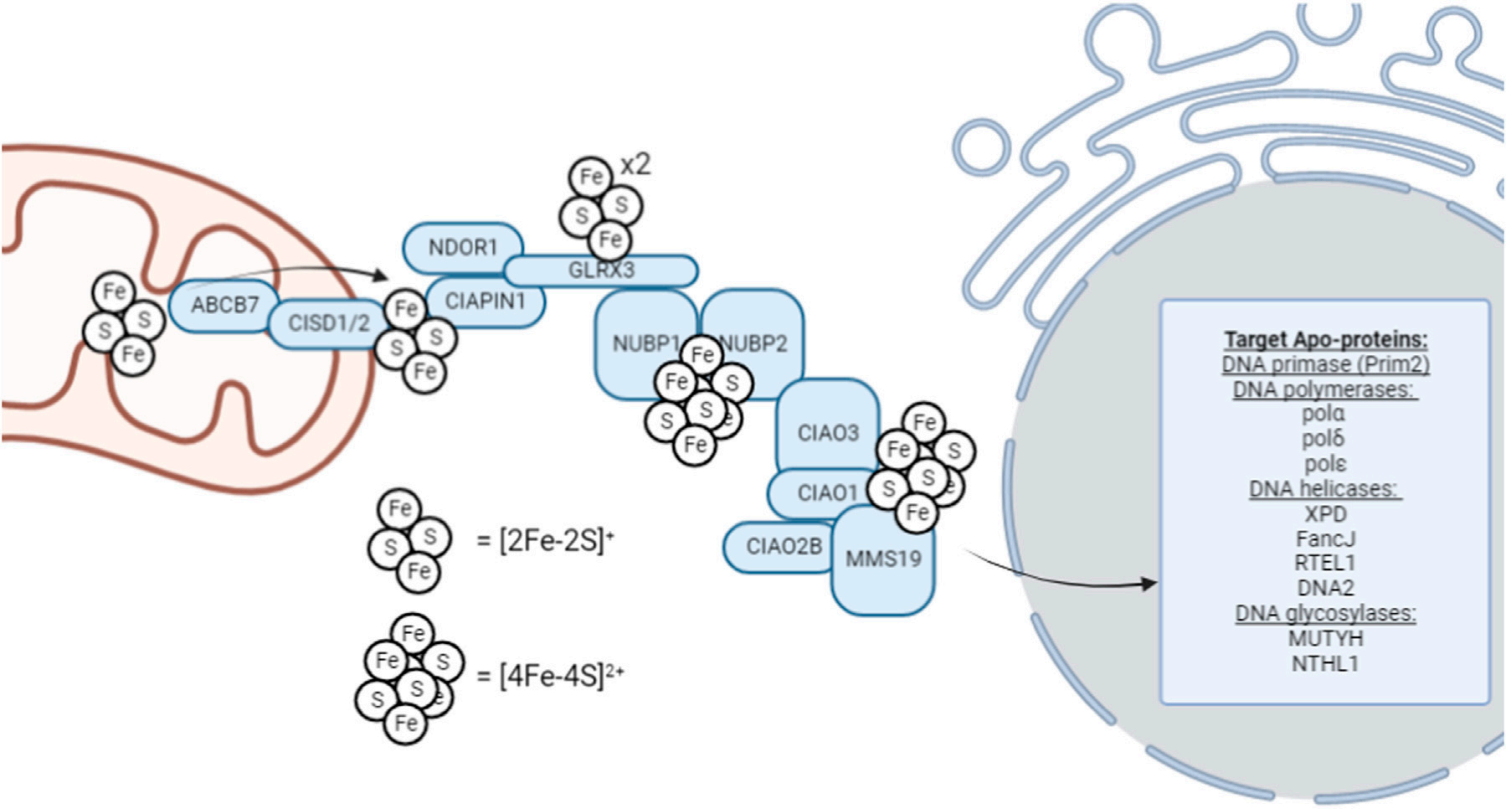
Schematic overview of the late-acting, cytosolic iron-sulfur assembly machinery (CIA complex). This schematic represents the general, proposed mechanism of [4Fe-4S]^2+^ cluster trafficking through the CIA complex for insertion into cytosolic and nuclear [4Fe-4S]^2+^ enzymes; however, not all factors are required definitively, and the utilization of this pathway can be highly context-dependent. For example, ribonucleotide reductase, critical for S-phase cell cycle entry to begin DNA replication, contains a di-ferric center whose formation is mediated by GLRX3 (Mühlenhoff et al., 2010).

Following completion of the *de novo* [2Fe-2S]^+^ cluster synthesis on the ISCU scaffold, [4Fe-4S]^2+^ formation and trafficking are required for insertion into DNA metabolic enzymes. Currently, the formation and trafficking of [4Fe-4S]^2+^ clusters and their biological implications are an active area of research and many of the connection points require further investigation. Trafficking of [4Fe-4S] clusters to cytosolic and nuclear apo-proteins are carried out by the cytosolic iron-sulfur assembly (CIA) pathway (Figure 1). This process begins with a necessary transfer of a [2Fe-2S]^+^ cluster from the mitochondria to the cytosol. This initial transfer is facilitated by ABCB7, a transmembrane protein that facilitates the transfer of the cluster out of the mitochondria (Stehling and Lill, 2013). Consistent with its proposed, exclusive role in the maturation of [4Fe-4S] clusters, ABCB7 deletion has little effect on mitochondrial [2Fe-2S]^+^ protein activity but results in functionally deficient cytosolic and nuclear [4Fe-4S]^2+^ proteins (Kispal et al., 1999; Pondarré et al., 2006; Miao et al., 2009). It has been proposed that a glutathione-coordinated [2Fe-2S]^+^ cluster ([2Fe-2S](SG)_4_) is the natural substrate for ABCB7, and is thus, represents the [2Fe-2S]^+^ cluster that is utilized by the CIA machinery (Qi et al., 2014; Li and Cowan, 2015) that allows for [4Fe-4S]^2+^ cluster formation to occur. However, there is still limited data regarding the mechanism of Fe-S transfer out of the mitochondria through ABCB7.

Cytosolic [4Fe-4S]^2+^ cluster biogenesis in eukaryotes is a complex process that remains an active area of investigation. This process is proposed to be initiated by the outer mitochondrial membrane-bound NEET proteins, which transfer the [2Fe-2S] cluster to the CIA assembly factors for MMS19-mediated insertion into target apo-proteins, however, this hypothesis remains an active area of research. MitoNEET (CISD1) and NAF-1 (CISD2) are [2Fe-2S]^+^ proteins located on the outer membrane of the mitochondria that aid in the maturation of extramitochondrial Fe-S proteins (Mittler et al., 2019). Both CISD1 and CISD2 can transfer their [2Fe-2S] cluster to anamorsin (CIAPIN1) of the CIA complex; however, their function has not been definitively elucidated (Lipper et al., 2015). The CIAPIN1/NDOR1 complex directly interacts with mitoNEET (CISD1) to reduce the [2Fe-2S]^+^ cluster (Camponeschi et al., 2017) as the flavorprotein NDOR1 uses an electron from NADPH to reduce the [2Fe-2S]^+^ cluster (Netz et al., 2010). This reduction step makes the cluster labile and thus provides a [2Fe-2S]^+^ cluster substrate is available for [4Fe-4S]^2+^ formation.

[4Fe-4S]^2+^ cluster formation occurs on the NUBP1-NUBP2 scaffold (Roy et al., 2003; Hausmann et al., 2005; Netz et al., 2012a; Pallesen et al., 2013) where the [2Fe-2S]^+^ cluster of the CIAPIN1/NDOR1 complex is transferred via GLRX3 to NUBP1-NUBP2 (Camponeschi et al., 2020). GLRX3 can bind two [2Fe-2S]^+^ clusters and thus, GLRX3-[2Fe-2S]_2_ can utilize glutathione to transfer the cluster to NUBP1 for [4Fe-4S]^2+^ cluster formation. NUBP1 and NUBP2 contain conserved cysteine residues at their C-terminal domain to coordinate a bridging [4Fe-4S]^2+^ cluster (Netz et al., 2012a). However, the [4Fe-4S]^2+^ cluster formed on the NUBP1-NUBP2 complex is also CIAO3-dependent (Balk et al., 2005). Similarly, CIAO3 is believed to be involved because it contains conserved cysteine motifs at its N- and C-terminal domains for Fe-S binding (Urzica et al., 2009).

Following the formation of a [4Fe-4S]^2+^ cluster on the NUBP1-NUBP2-CIAO3 complex, it can be transferred to the appropriate apo-proteins by the late-acting CIA complex. This process occurs through MMS19. MMS19 is able to form a complex with CIOA1, CIAO2B, AND CIAO3 to make up the CIA targeting complex (Gari et al., 2012). The completed [4Fe-4S]^2+^ cluster is hypothesized to transferred to the CIA targeting complex (CIAO1, CIAO2B, and MMS19) by CIAO3 (Kassube and Thomä, 2020), but further data is required to illuminate the role of CIAO3 as a mediator of cluster transfer. CIAO2B and CIAO1 associate with the C-terminus of MMS19 to form a docking site for cytosolic and nuclear apo-proteins, however XPD has been observed to directly interact with MMS19 (Odermatt and Gari, 2017). Interestingly, MMS19 binding prevents CIAO2B proteasomal degradation as an apparent feedback regulatory mechanism to maintain CIA stability (Seki et al., 2013).

When considering the regulation of DNA metabolism, MMS19 is the main connection point as MMS19 serves as a scaffold for the transfer of the completed [4Fe-4S] cluster to the appropriate apo-protein. MMS19 has a docking site that allows it to directly interact with [4Fe-4S]^2+^ containing DNA metabolic proteins necessary for maintaining genomic stability (Gari et al., 2012). To underscore the importance of MMS19 as a regulatory component of DNA metabolism, MMS19 knockdown has been observed to result in decreased XPD, FANCJ, and DNA polymerase expression. Furthermore, MMS19 depletion strongly decreases the expression of the POLD1 subunit of DNA polymerase *δ* (Stehling et al., 2012). Thus, it appears that the late-acting CIA-complex, culminating in the insertion of a [4Fe-4S]^2+^ cluster into nuclear apo-proteins using MMS19 as a scaffold, is a critical regulatory step in ensuring that [4Fe-4S]^2+^ cluster-containing DNA metabolic enzymes are functional to aid in the maintenance of genomic integrity through DNA replication.

## Iron-sulfur clusters in DNA metabolism

For DNA to be efficiently and accurately passed to progeny cells, high-fidelity DNA replication is required. At each level of maintaining genome integrity, Fe-S-containing enzymes are necessary for this process to occur (Table 1). Mechanistically, it is hypothesized that the [4Fe-4S]^2+^ clusters serve as electrochemical sensors that can detect electron transport along the DNA backbone. DNA charge transfer occurs when electrons are passed through the pi-stack of base pairs between redox partners (Boal et al., 2009; Slinker et al., 2011). DNA charge transfer can occur in intact double-stranded DNA, but any disruption to the base pair stacking (e.g., DNA base damage, DNA strand break) will disrupt this process. Thus, intact double-stranded DNA can be theoretically considered a wire that allows electrons to move along the strand and impediments to this electron movement will allow for the identification and reparation of damage. When bound to DNA, the [4Fe-4S]^2+^ cluster contained within proteins have a redox potential of ≈ −200 mV, which allows it to serve as a redox switch by cycling between a [4Fe-4S]^2+/3+^ oxidation state as electrons move along the double-stranded DNA (Fuss et al., 2015). In this context, the [4Fe-4S] cluster may be critical feature of key DNA metabolic enzymes that can serve as both an electron donor or acceptor, allowing it to function as a redox sensor of DNA damage via cluster oxidation following electron transport along the DNA backbone (Fuss et al., 2015; Arnold et al., 2016; Syed and Tainer, 2019). Furthermore, we describe the function of the various [4Fe-4S] containing enzymes and their role in DNA metabolism.

**TABLE 1:** [4Fe-4S] cluster enzymes involved in DNA metabolism.

Enzyme	Function	[4Fe-4S] cluster (subunit)	Involvement in DNA metabolism	References
POLA	Catalytic subunit of DNA polymerase α	C-terminal domain (catalytic subunit)	Replication fork extension	Netz et al. (2012b), Kilkenny et al. (2012)
POLD1	Catalytic subunit of DNA polymerase δ	C-terminal domain (catalytic subunit)	Replication fork extension	Netz et al. (2012b), Jozwiakowski et al. (2019)
POLE1	Catalytic subunit of DNA polymerase ε	C-terminal domain (catalytic subunit)	Replication fork extension	Netz et al. (2012b), ter Beek et al. (2019)
XPD	Helicase	N-terminal domain (catalytic subunit)	Subunit of transcription initiation factor TFIIH; nucleotide excision repair, DNA damage recognition	Rudolf et al. (2006)
FANCJ	Helicase	N-terminal domain (catalytic subunit)	DNA secondary structure resolution (e.g., G-quadruplex, G4)	Rudolf et al. (2006)
RTEL1	Helicase	Crystal structure unresolved	Telomere maintenance, DNA secondary structure resolution (e.g., G-quadruplex, G4); Homologous recombination, D-loop resolution	Uringa et al. (2010)
DNA2	Helicase	Nuclease active site	DNA replication; dsDNA break repair	Pokharel and Campbell (2012)
DDX11/CHLR1	Helicase	Helicase domain	Sister chromatid cohesion; DNA secondary structure resolution (e.g., G-quadruplex, G2′)	Simon et al. (2020)
PRIM2	DNA primase	p58C domain	DNA replication initiation; DNA synthesis; dsDNA damage repair	O’Brien et al. (2017)
MUTYH	DNA glycosylase	Fe-S loop adjacent to N-terminal—C-terminal connection	Base excision repair	Guan et al. (1998)
NTHL1	DNA glycosylase	N-terminal—C-terminal connection	Base excision repair	Carroll et al. (2021)

### DNA helicases

For DNA to be replicated, the double-stranded DNA must first be opened by helicases. Helicases are motor proteins that utilize ATP hydrolysis to translocate along and unwind the paired nucleic acids that make up double-stranded DNA. Due to the complexities of double-stranded DNA maintenance, helicases are largely responsible for regulating several different processes of DNA separation (e.g., DNA replication/repair and telomere regulation) (Abdelhaleem, 2010). Thus, helicases are critical features of nucleic acid metabolism and maintaining genome stability. Six superfamilies of helicases are designated based on their amino acid sequence with several containing [4Fe-4S] clusters that are required for their functioning (Singleton et al., 2007). Each of these helicases are linked to the CIA complex through interaction with MMS19 that delivers the completed [4Fe-4S] cluster (Stehling et al., 2012).

DNA2 is an Fe-S containing member of helicase superfamily 1 involved in DNA replication, telomere maintenance, and double-strand break (DSB) repair. Thus, DNA2 serves a central role in maintaining genome stability at multiple phases (Budd et al., 2005; Zheng et al., 2020). Functionally, DNA2 has helicase and nuclease activity, which depend on the presence of a [4Fe-4S] cluster (Pokharel and Campbell, 2012). The [4Fe-4S] cluster of DNA2 is bound to four cysteine residues contained within the nuclease domain, suggesting that the cluster may serve to maintain the structural integrity of the nuclease domain, thus, rendering it essential for facilitating DNA2 binding of broken DNA (Yeeles et al., 2009). A 2020 study by Mariotti et al. (2020) showed that loss of the [4Fe-4S] cluster caused a conformational change in DNA2 resulting in a distortion of the central DNA binding tunnel. This study also showed that oxidation of DNA2 impaired DNA binding *in vitro* that was reversible by reduction; however, this effect was independent of the presence of the [4Fe-4S] cluster. Thus, DNA2 represents an example of a nuclear enzyme where the [4Fe-4S] protein plays a critical structural role.

Helicase superfamily 2 also encompasses several Fe-S containing members. A commonly recognized Fe-S containing helicase is XPD. XPD is a part of the TFIIH complex that is involved in DNA transcription and unwinding dsDNA for damage verification and initiation of nucleotide excision repair (Houten et al., 2016). Interestingly, a 2014 study by Kuper, et al. showed that the enzymatic activity of XPD within the TFIIH complex is mainly dedicated to DNA damage recognition and resolution, while it primarily functions to maintain the structural integrity of the TFIIH complex during transcription initiation (Kuper et al., 2014). XPD contains a [4Fe-4S] cluster within its catalytic domain that is thought to play a structural role but may be necessary in DNA damage recognition (Wolski et al., 2008; White, 2009). XPD has been observed to interact with the CIA complex and the TFIIH complex in a mutually exclusive fashion suggesting that the [4Fe-4S] cluster of XPD is first inserted in the cytoplasm by the CIA complex before translocation to the nucleus for its association with the TFIIH complex (Vashisht et al., 2015). XPD with deficient Fe-S binding or impaired CIA interaction was unable to join the TFIIH complex and MMS19 deletion causes a depletion of XPD (Kou et al., 2008; Vashisht et al., 2015). XPD can be linked to three separate genetic disorders: xeroderma pigmentosum, Cockayne syndrome, and trichothiodystrophy which can be linked to various mutations in XPD (Taylor et al., 1997). Xeroderma pigmentosum and Cockayne syndrome mutations impair the Fe-S binding domain and impair helicase activity, while trichothiodystrophy mutants have been observed in all four XPD domains to impair the XPD secondary structure likely leading to impaired TFIIH integrity (Fan et al., 2008). Thus, xeroderma pigmentosum and Cockayne syndrome are considered DNA repair related disorders while trichothiodystrophy is due to impaired transcription. Overall, it appears that the insertion of a [4Fe-4S] cluster into XPD is essential for its ability to function within the TFIIH complex to promote efficient DNA damage repair.

Another Fe-S containing superfamily 2 helicase is FANCJ (Brosh and Cantor, 2014). FANCJ is a helicase that functions in double-stranded DNA damage repair through homologous recombination and can resolve DNA secondary structures to promote smooth DNA replication and the avoidance of replication stress (Datta and Brosh, 2019). FANCJ was initially discovered as a result of a physical interaction with the renowned tumor suppressor gene, BRCA1, as it binds at the BRCT motifs of BRCA1 (Cantor et al., 2001). In this seminal report, the FANCJ/BRCA1 complex (initially referred to as BRCA1 interacting C-terminal helicase, BACH1), was shown to be important for the DNA damage response function of BRCA1. In addition to FANCJ, BRCA1 can also associate with a non-Fe-S helicase FANCM, that has been shown to be essential to mitigate replication fork stalling and mediate D-loop dissociations (Gari et al., 2008; Panday et al., 2021). This underscores the importance of the interaction of BRCA1 and the Fancomi family of helicases in the maintenance of genome integrity. Beyond its interaction with BRCA1, FANCJ functions as an ATP-dependent helicase with 5′-3′ specificity that requires an intact [4Fe-4S] cluster, similar to XPD (Rudolf et al., 2006). More specifically, FANCJ is a helicase that can unwind DNA G-quadruplexes ahead of DNA polymerase, which are guanine-rich DNA secondary structures that cause DNA replication stalling and are prone to oxidative damage (London et al., 2008; Wu and Spies, 2016; Lerner and Sale, 2019; Fleming and Burrows, 2021). Also, like XPD, FANCJ has been shown to directly interact with that late acting CIA complex through MMS19 (Wietmarschen et al., 2012) as MMS19 knockdown significantly impairs FANCJ iron insertion to promote genomic instability and sensitivity to DNA damaging agents (Weon et al., 2017). Thus, the insertion of the [4Fe-4S] cluster into FANCJ is a critical step in its ability to preserve genomic integrity through its helicase function.

The third Fe-S containing member of the helicase superfamily 2 is DDX11/CHLR1. Similar to both XPD and FANCJ, DDX11 is an ATP-dependent helicase with 5′-3′ directionality with a preferred single stranded 5′ tail (Hirota and Lahti, 2000; Farina et al., 2008). The helicase function of DDX11 allows it to serve a similar role to FANCJ in the resolution of G-quadruplexes to prevent replication stress (Wu et al., 2012; Bharti et al., 2013; van Schie JJMFaramarz et al., 2020). However, unlike FANCJ, which can efficiently resolve unimolecular (G4) G-quadruplexes (Wu and Spies, 2016), DDX11 efficiently unwinds two stranded anti-parallel (G2′) G-quadruplexes to a much greater extent than G4 structures (Wu et al., 2012). Consistent with its role in maintaining genome integrity, DDX11 depletion has been shown to decrease the amount of single-stranded DNA leading to impaired CHK1 phosphorylation (Simon et al., 2020), a critical step in the DNA damage response pathway, promoting DNA replication stress (Patil et al., 2013; Jegadesan and Branzei, 2021). Importantly, the [4Fe-4S] cluster of DDX11 is indispensable for its functionality in the resolution of DNA secondary structures to prevent replication stress (Simon et al., 2020). DDX11 is primarily recognized for its role in maintaining sister chromatid cohesion as an inactivating mutation results in the cohesinopathy called Warsaw Breakage Syndrome (van der Lelij et al., 2010; Capo-Chichi et al., 2013; Bharti et al., 2014). The DDX11 variant associated with Warsaw Breakage Syndrome (R263Q) cannot bind an Fe-S cluster (Simon et al., 2020). Thus, DDX11 represents a helicase where the [4Fe-4S] cluster is critical to its enzymatic activity.

The final Fe-S containing member of the helicase superfamily 2 is RTEL. RTEL is a [4Fe-4S] cluster critical for maintaining genome stability through telomere maintenance and double-stranded DNA damage repair (Uringa et al., 2010). RTEL has been shown to play a critical role in setting telomere length in mice (Ding et al., 2004) and suppression of RTEL in mouse embryonic fibroblasts results in increased telomere fragility (Sfeir et al., 2009). Moreover, it has been shown that RTEL is required for telomere replication in mouse embryonic fibroblasts. RTEL depletion increases G4 stability at telomeres to prevent telomere replication (Uringa et al., 2012). This is consistent with biochemical data showing that RTEL can resolve G-quadruplexes to prevent replication and promote telomere lengthening throughout the human genome (Wu et al., 2020). Beyond telomere maintenance, RTEL has been shown to play an important role in DNA damage repair (Uringa et al., 2010). RTEL has been shown in *C. elegans* to regulate homologous recombination and promote genomic stability by resolving D-loops where RTEL mutants showed an increased propensity to accumulate DNA damage (Barber et al., 2008). Currently, the role of the [4Fe-4S] cluster in RTEL (i.e., structural versus functional) remains unclear; however, similar to the other Fe-S containing helicases, RTEL does interact directly with MMS19 (Stehling et al., 2012).

### DNA primase and polymerases

Following the opening of a double stranded DNA helix by helicases, replication can occur by DNA polymerases. However, DNA polymerases are unable to initiate the synthesis of a new DNA strand during replication, rather they only extend existing strands and thus, require a primer. DNA primase is the enzyme responsible for synthesizing a short primer for DNA polymerase to use as a template (Frick and Richardson, 2001). Similar to the Fe-S containing helicases, DNA primase acquires a [4Fe-4S] cluster from MMS19 and the functionality of DNA primase is dependent on an intact [4Fe-4S] cluster (Klinge et al., 2007; Stehling et al., 2012). It has been observed that the [4Fe-4S] cluster of DNA primase serves as a redox switch that modulates its DNA binding capacity (Holt et al., 2017). DNA primase is a heterodimer with a small and large subunit with the small subunit being responsible for RNA polymerase activity and the large [4Fe-4S] subunit (PRIM2) being responsible for DNA binding (Agarkar et al., 2011). The Fe-S cluster has a [4Fe-4S]^2+^ resting state where it is loosely bound to DNA, however, an oxidation of the cluster to a [4Fe-4S]^3+^ state results in tight DNA binding (O’Brien et al., 2017; O’Brien et al., 2018a; O’Brien et al., 2018b). Thus, it appears that Fe-S mediated DNA charge transfer is an essential feature of DNA replication initiation mediated by PRIM2.

Following the generation of a short primer by DNA primase, DNA is replicated by [4Fe-4S] containing DNA polymerases. In eukaryotic cells, class B family DNA polymerases *α*, *δ*, *ε*, and *ζ* mediate DNA replication. All three of these polymerases contain a [4Fe-4S] within their C-terminal catalytic subunits (POLA, POLD1, and POLE1, respectively) (Garcia-Diaz and Bebenek, 2007; Shi et al., 2021). It was believed that Zn^2+^ was the necessary inorganic co-factor for polymerase activity due to the two conserved cysteine residues acting as metal binding motifs in POLA (Evanics et al., 2003; Klinge et al., 2009). However, later structural experiments revealed that all four polymerases coordinate a [4Fe-4S] cluster within the catalytic subunit (Netz et al., 2012b; ter Beek et al., 2019; Suwa et al., 2015; Baranovskiy et al., 2018). Loss of the [4Fe-4S] cluster in POLD1 causes a destabilization of all four enzyme subunits resulting in defective DNA binding and impaired polymerase and exonuclease activities (Jozwiakowski et al., 2019). In yeast, the [4Fe-4S] cluster of DNA polymerase ε is redox active and its polymerase function may be mediated by DNA charge transfer similar to PRIM2 (Jain et al., 2013; Pinto et al., 2021). Consistent with other DNA metabolic enzymes, the assembly of the [4Fe-4S] cluster in the catalytic subunit of DNA polymerases and ultimately their enzymatic activity are mediated by MMS19 of the CIA complex (Gari et al., 2012; Stehling et al., 2012; Han et al., 2015). Therefore, it is apparent that maintenance of high-fidelity DNA replication is largely dependent on the insertion of completed [4Fe-4S] clusters into the appropriate DNA metabolic enzymes by the late acting CIA complex.

### DNA glycosylases

While DNA helicases and polymerases aim to perform high-fidelity DNA replication to avoid replication stress, damage to DNA bases can occur through several different chemical modifications including oxidation, alkylation, deamination, and spontaneous hydrolysis (Bauer et al., 2015). The primary enzymatic pathway for the repair of damaged DNA bases is base excision repair (BER), which can occur throughout the cell cycle (Krokan and Bjoras, 2013). BER is initiated by damage recognition by DNA glycosylases which then form AP sites to remove the damaged bases (Jacobs and Schär, 2012; Wallace, 2013). Short patch or long patch base excision repair can occur based on the number of damaged bases. Two DNA glycosylases with [4Fe-4S] clusters have been identified, MUTYH and NTHL1 (Parker et al., 2000; Carroll et al., 2021). Both MUTYH and NTHL1 are mammalian MutY and endonuclease III homologs, *E. coli* DNA glycosylases that were initially observed coordinate a [4Fe-4S] cluster that mediates their DNA binding and substrate recognition (Kuo et al., 1992; Guan et al., 1998). MUTYH is an adenine-specific glycosylase that removes mismatched adenines from A-G/A-C pairs and can also remove 8-dihydro-8-oxodeoxyguanine (8-oxoG) (McGoldrick et al., 1995). Meanwhile, NTHL1 can excise thymine glycol and oxidize urea lesions (Aspinwall et al., 1997). The [4Fe-4S] cluster of MutY and endonuclease are redox active and serve as an electrochemical sensor to recognize DNA damage through DNA charge transfer (Boal et al., 2005). Thus, it furthers the hypothesis that the [4Fe-4S] clusters of DNA regulatory enzymes serve as conserved sensors of DNA charge transfer to efficiently maintain genomic integrity.

## Conclusion and future perspectives

With the current understanding that several [4Fe-4S] cluster enzymes interact with the late-acting CIA complex and play a critical role in DNA metabolism, there is a window of opportunity to accelerate our understanding of how Fe-S biogenesis can regulate metabolic processes (e.g., maintain genomic integrity). As the biomedical community works to understand the various systems that play a role in regulating health and disease, the regulatory role of Fe-S biogenesis remains unclear. However, a wide array of literature suggests that Fe-S biogenesis plays a role in numerous diseases which may result from the dysregulated global metabolic issues that arise from disrupted Fe-S containing enzymes such as those described in this review. For example, mutations associated with the [4Fe-4S] containing helicases have been implicated in the onset of disease as XPD mutations present as xeroderma pigmentosum, Cockayne syndrome, and trichothiodystrophy (Taylor et al., 1997); FANCJ as Fancomi Anemia (London et al., 2008); DDX11 as Warsaw Breakage Syndrome (van der Lelij et al., 2010; van Schie JJMFaramarz et al., 2020); DNA2 as mitochondrial DNA depletion syndrome (Sun et al., 2022); and RTEL mutations have been associated with familial pulmonary fibrosis (Kannengiesser et al., 2015). Additionally, a majority of these enzymes are associated with cancer development including XPD, FANCJ, DDX11, RTEL, MUTYH, and NTHL1 (Benhamou and Sarasin, 2002; Nicolo et al., 2008; Lubbe et al., 2009; Cantor and Guillemette, 2011; Mazzei et al., 2013; Weren et al., 2015; Yan et al., 2016; Das et al., 2020; Hutchcraft et al., 2021; Magrin et al., 2021; Mahtab et al., 2021).

Following the discovery that MMS19 directly interacts with enzymes that regulate the maintenance of genome integrity and high-fidelity transfer of genetic information including helicases, primase, polymerases, and glycosylases, there is a very clear connection between Fe-S biogenesis through the late-acting CIA complex and DNA metabolism (Gari et al., 2012; Stehling et al., 2012). While the insertion of a complete [4Fe-4S] cluster by MMS19 into these DNA metabolic enzymes represents a critical regulatory step for the maintenance of genomic integrity; it is important to acknowledge the several steps leading to the formation of the completed [4Fe-4S]^2+^ cluster on MMS19 before the insertion into nuclear enzymes (Petronek et al., 2021). Thus, the transfer of the cluster from the CIA complex through MMS19 to nuclear enzymes represents the penultimate step in a larger process that, if impaired, likely results in genome instability. For example, frataxin loss from *de novo* [2Fe-2S] cluster synthesis results in Friedrich’s Ataxia and predisposes cells to DNA damage associated with impaired BER, a process that is initiated by [4Fe-4S] containing DNA glycosylases (Haugen et al., 2010; Thierbach et al., 2010; Shen et al., 2016). Thus, it may be imperative to consider the implications of changes in Fe-S biogenesis in totality when investigating global, cellular metabolic alterations in various pathologies associated with genomic instability.
